# Partners in Crime: Towards New Ways of Targeting Calcium Channels

**DOI:** 10.3390/ijms20246344

**Published:** 2019-12-16

**Authors:** Lucile Noyer, Loic Lemonnier, Pascal Mariot, Dimitra Gkika

**Affiliations:** 1 Univ. Lille, Inserm, U1003-PHYCEL-Physiologie Cellulaire, F-59000 Lille, France; lucile.noyer@inserm.fr (L.N.); loic.lemonnier@inserm.fr (L.L.); pascal.mariot@univ-lille.fr (P.M.); 2 Laboratory of Excellence, Ion Channels Science and Therapeutics, Université de Lille, 59655 Villeneuve d’Ascq, France

**Keywords:** TRP channel, CaV channel, domain interactions, TCAF, Rap, IP3R, sigma receptor

## Abstract

The characterization of calcium channel interactome in the last decades opened a new way of perceiving ion channel function and regulation. Partner proteins of ion channels can now be considered as major components of the calcium homeostatic mechanisms, while the reinforcement or disruption of their interaction with the channel units now represents an attractive target in research and therapeutics. In this review we will focus on the targeting of calcium channel partner proteins in order to act on the channel activity, and on its consequences for cell and organism physiology. Given the recent advances in the partner proteins’ identification, characterization, as well as in the resolution of their interaction domain structures, we will develop the latest findings on the interacting proteins of the following channels: voltage-dependent calcium channels, transient receptor potential and ORAI channels, and inositol 1,4,5-trisphosphate receptor.

## 1. Introduction

Calcium transport through biological membranes underpins numerous fundamental physiological processes such as muscular contraction, electrical signaling in the heart and the nervous system, as well as hormone secretion, immune response, cell migration, and cell growth [1,2]. The biological significance of calcium channels is underlined by their involvement in a wide variety of pathologies spanning all the major areas of therapeutic interest affecting the cardiac, neuronal, neuromuscular, neurosensory, metabolic, epithelial, and respiratory systems. Given their diverse biological roles, it is not surprising that calcium channels form a major class of drug targets acting mainly on the channel gating [3,4,5,6].

However, calcium flux through the membrane is not solely determined by the biophysical properties of the oligomeric channel pore but also by the cross-talk of this later with accessory proteins such as adaptors, scaffolds, and regulators. The calcium channel interactome appears to involve a highly diverse array of molecular interactions, ranging from tight physical association to rather loose and dynamic interactions [7,8]. This huge structural diversity is a major challenge for molecular drug discovery. In many cases, the exact interactome composition of a target channel in the tissue of interest is only poorly characterized, and the functional roles of the channel interacting proteins are not well defined. Another challenge is the very dynamic behavior of ion channels with their interacting partners that ultimately determines the macroscopic currents at the plasma membrane.

Taking into account the recent advances in the growing field of partner protein regulation of ion channels, we will present here the functional interactions with the following calcium channels: voltage-dependent calcium channels (VDCC), transient receptor potential (TRP) and store-operated calcium (SOC) channels, and the inositol 1,4,5-trisphosphate receptor (IP3R).

## 2. Targeting Voltage-Dependent Calcium Channel Interactions

VDCC are all activated by a membrane depolarization but differ by their electrophysiological and pharmacological properties, which allows them to be classified in different subclasses: high-voltage activated channels (HVA) or low-voltage activated (LVA). HVA channels are activated by a large depolarization (from −30 mV) and are subdivided in L, N, P/Q, and R channels according to their sensitivity to dihydropyridines (DHP) and other inhibitors [9,10,11]. LVA channels, also called T-type channels, are activated by a small depolarization (between −80 and −60 mV) and are characterized by a tiny conductance, a transient opening, and a high sensitivity to Ni^2+^ blockade [12,13,14]. The essential component of VDCC is the alpha1 (α1) subunit (also called CaVx.y subunits). To date 10 different α1 subunits have been characterized and shown to constitute the pore subunits for L-type channels (CaV1.1, CaV1.2, CaV1.3, and CaV1.4), P/Q, N, and R channels (CaV2.1, CaV2.2, and CaV2.3), and T-type channels (CaV3.1, CaV3.2, and CaV3.3) [15]. These α1 subunits are responsible for the main electrophysiological and pharmacological properties and form a functional complex with accessory or regulatory subunits (β, γ, and α2-δ) for HVA channels, whereas LVA channels do not require accessory subunits and can be constituted only by the pore subunits [8]. In addition to the aforementioned accessory subunits, there are numerous proteins which interact with VDCC α1 pore subunits, among which calmodulin [16] various GTPases [17] members of the synaptic machinery (SNAP25, syntaxin 1A and synaptotagmin I interacting with CaV2.1 or CaV2.2 for example [18,19] and other ion channels (such as potassium channels [20]). We have chosen to specifically address in this section VDCC interactions with proteins partners (β2 subunits, Galectin-1, Kir/Gem GTPases, CRMP2, and USP5) that have been successfully targeted with specific peptides when these interactions were shown to be responsible for the onset of diseases or disorders.

VDCC channels are involved in many aspects of cell physiology in excitable tissues, from excitation-contraction coupling in muscle cells, neurotransmitter release in presynaptic buttons, hormone release by endocrine cells, and bursting activity in neurons. As a consequence, VDCC are a common target for treating neuromuscular, pain, and cardiovascular diseases [6,21,22]. A great number of drugs have been therefore developed over the last decades to block VDCC, such as dihydropyridines (nifedipin), phenylalkylamines (verapamil), benzothiazepin (diltiazem), flunarizin, and gabapentinoids (gabapentin, pregabalin) (for a review see [6]). However, all these drugs are associated with side-effects. As exemplified below in several examples, various studies using permeant peptides have thus attempted to specifically interfere with the interaction between pore and accessory subunits in order to either stimulate or inhibit VDCC activity.

Heart ischemia-reperfusion injuries are responsible for cardiac failures due to multiple cell metabolism disorders occurring during superfusion among which are increased reactive oxygen species (ROS) production and high cytosolic calcium concentrations. Since L-type channels are the main pathways for calcium entry into cardiac myocytes, calcium antagonists have been used for many years to treat ischemia [23]. It was proposed that specifically targeting VDCC function may be decisive for alleviating ischemia-superfusion disorders [24]. In HVA channels, the interaction between cytoplasmic β and transmembrane α1 subunits occurs through specific amino-acid sequences, the AID (α1 Interaction Domain) an 18 amino acid sequence in the intracellular loop connecting the domain I and II, in the α1 subunit and a region named the ABP (for AID-Binding Pocket) located in a guanylate kinase (GK) domain of the β subunit (for a review see [25]). Serving as chaperones, β subunits promote the expression, and modify the electrophysiological properties, of VDCC at the plasma membrane [8]. It was shown that synthetic AID peptides antagonized the increase in open probability of CaV1.2 channels promoted by β2 subunits, thereby reducing calcium entry [26]. Several studies [24,27] showed that delivery of AID synthetic peptides in guinea-pig cardiac myocytes reduced ischemia-reperfusion injuries as revealed by a reduction in cytosolic calcium concentration, in creatine kinase and lactate dehydrogenase. This was achieved either with both TAT (trans activator of transcription)-bound AID peptides or AID loaded nanoparticles [24]. Using stapled AID peptides, it was shown recently that these synthetic peptides inhibited calcium channels through an inhibition of CaV1.2 protein–β2 protein interaction [28] providing a proof of concept for using synthetic peptides to inhibit subunit association and VDCC activity.

Interestingly, in a number of cardiac pathologies, there is a decreased expression of CaV1.2 VDCC [29]. A recent study has demonstrated that the physiological expression of CaV1.2 at the plasma membrane depends on Akt-dependent phosphorylation of β2 at its C-terminus, allowing binding of β2 with CaV1.2 [30]. Another actor, Kir/Gem, also regulates the association between CaV1.2 and β subunits. This protein, a member of RGK small GTP-binding protein family, has been shown to interact with SH3 domain of β subunits on and prevents their association with CaV1.2, thereby impeding the expression of VDCC at the plasma membrane [31,32]. Rusconi et al. [30] developed a mimetic peptide (MP) fused with an oligo Arginine cell permeant peptide (RW7-MP). They showed that, through an interaction with the SH3 domain of β2 subunits, thus competing with Kir/Gem, the RW7-MP could restore the expression of VDCC at the plasma membrane of cardiomyocytes in a model of diabetic mice [30].

In smooth muscle, CaV1.2 is the main regulator of calcium entry responsible for myocyte contraction and its overexpression is usually associated with hypertension [33,34]. It was shown recently that Galectin-1 (Gal-1) interacts with the intracellular I-II loop of CaV1.2 channels and competes for this site with β2 subunits [35]. The disruption of CaV1.2–β2 interaction by Gal-1 leads to CaV1.2 degradation by the proteasome. The authors demonstrated in the same study that injection of Tat-peptides competing with Gal-1 decreased CaV1.2 degradation and induced hypertension in rats.

VDCC and most particularly Cav2.2 are important actors in neuropathic pain transduction. Inhibitors of VDCC are thus used to treat neuropathic pain, such as Prialt^®^ [5,36], a synthetic version of µ-conotoxin MVIIA which blocks CaV2.2, or most commonly gabapentinoids like gabapentin or pregabalin [37] which inhibit α2-δ partner protein. CRMP2 (collapsin response mediator protein 2), another partner protein of VDCC (for a review see [38]), has been shown to interact specifically with CaV2.2 and no other VDCC [39] to promote its trafficking and to increase calcium current density [38]. Based on the structure of the calcium channel binding domain (CBD) of CRMP2, CBD peptides have been developed to disrupt CRMP2 interaction with the I-III loop of CaV2.2. Among these peptides, one (CBD3) was more effective to reduce CaV2.2 mediated currents and when injected as a TAT-CBD3 peptide, was able to reduce pain behaviors (for a review see [38]). A recent study demonstrated that delivery of CBD3 peptides using recombinant adeno-associated virus (AAV) is particularly efficient to attenuate pain behavior [40].

CaV3.2, as CaV2.2, is a key player in nociception. It has been shown to be upregulated in various painful diseases and its pharmacological inhibition induces analgesia. It was recently demonstrated that this nociceptive effect is due to an increase in channel activity, which is primarily mediated by an upregulation of USP5, a deubiquitinating enzyme, which associates with CaV3.2 and inhibits its degradation [41]. Using an intrathecal injection of a permeant peptide which impairs the interaction between USP5 and CaV3.2, several studies showed that it was possible to relieve rapidly (in a few minutes) pain sensation related to mechanical or thermal hypersensitivity.

**Figure 1:**
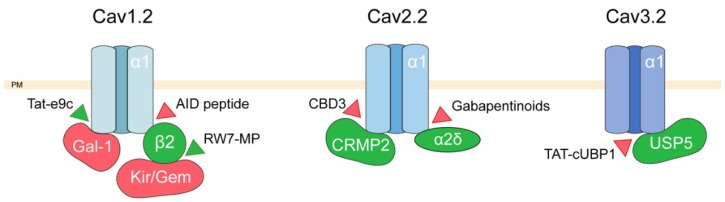
Targeting voltage-dependent calcium channels channel. Scheme depicting Cav1.1, Cav2.2, and Cav3.2 channels interactions with their activating (in green) and inhibiting (in red) partner proteins. Molecules targeting these interactions are represented by triangles: red triangles indicate an inhibitory effect, green triangles an activating effect. PM, plasma membrane; CRMP2, collapsin response mediator protein 2; Gal-1, Galectin-1, USP5 deubiquitinating enzyme; β2 regulatory subunit; α2-δ regulatory subunit.

As a conclusion, many approaches using permeant peptides but also nonpeptidic synthetic molecules [42,43] have managed to alter the interaction between voltage-dependent calcium channel partners in order to successfully reverse various symptoms associated with different pathologies. These interactions are summarized in Figure 1.

## 3. TRP Channel Interactors

**Figure 2:**
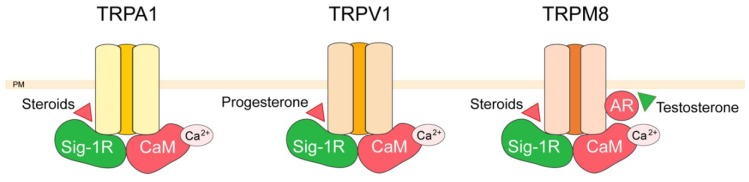
Targeting transient receptor potential channels. Scheme depicting TRPA1, TRPV1, and TRPM8 channels and their partner proteins. Sigma-1 receptor (Sig-1R) activating partner protein is shown in green and inhibiting partner proteins such as calmodulin (CaM) and androgen receptor (AR) is shown in red. Steroids targeting these interactions are represented by triangles: red triangles indicate an inhibitory effect, green triangles an activating effect. PM, plasma membrane.

TRP channels form a relatively new ion channel family, of 30 years old, and were first described in Drosophila, where photoreceptors carrying *trp* gene mutations exhibited a transient voltage response to continuous light [44]. Since then, about 30 TRPs were identified which are classified in six different families: TRPC (Canonical), TRPV (Vanilloid), TRPM (Melastatin), TRPML (Mucolipin), TRPP (Polycystin), and TRPA (Ankyrin transmembrane protein) [45]. Unlike most ion channels, TRP channels are identified by their homology rather than by ligand function or selectivity, even though they display poor sequence identity as low as 20%. TRP channels are putative six-transmembrane (6TM) polypeptide subunits that assemble as tetramers to form cation-permeable pores. All functionally characterized TRP channels are permeable to calcium with the exceptions of TRPM4 and TRPM5, which are only permeable to monovalent cations. Most calcium permeable TRP channels are only poorly selective for calcium, with a permeability ratio relative to sodium (PCa/PNa) in the range between 0.3 and 10. Exceptions are TRPV5 and TRPV6, two highly Ca^2+^-selective TRP channels with PCa/PNa = 100 [46]. The members of the TRP channel super-family are regarded as cellular sensors [47] integrating external and endogenous stimuli, and thus contribute to cell-to-cell communication and maintain many forms of homeostasis. TRP channels are associated with several pathophysiological processes, which include (but are not limited to) diseases linked to pain, cardiovascular function, pulmonary function, inflammation, skin, neurological disorders, kidney, bone, obesity, as well as proliferative diseases via dysregulation of the cell cycle, carcinogenesis, and tumor angiogenesis [40,41,42]. TRP channels therefore serve as communication hubs for the cells that express them, and their regulation is crucial for their function. Several partner proteins have been identified for TRPs over the last decades, among which some are common for different members of this channel family. Currently 700 of the protein–protein interactions of TRP channels are gathered and visualized in network database [48]. Among these interactions, we will focus here on the relatively recent field of nongenomic action of steroids on TRP channels. In this regard we will describe how protein–protein interaction defines the hormonal action on TRP channel activity via the Sigma-1 (Sig-1R) and the androgen receptor as depicted in Figure 2.

The Sig-1R is a protein mainly localized in the endoplasmic reticulum, where it functions as a ligand-operated chaperone [49]. Upon agonist binding Sig-1R translocates at the plasma membrane where it interacts with ion channels [50,51,52,53]. Many factors among which steroids act on the Sig-1R resulting in negative or positive effects on the function and plasma membrane expression of potassium, calcium, and TRP channels [54]. Sig-1R was described to interact with TRPV1, TRPA1, TRPM8 in calcium and ligand-dependent ways [55,56]. Sig-1R and calmodulin were shown to bind directly to cytosolic regions of these three TRPs, and that this binding increasing in the presence of calcium. Sig-1R prevents calmodulin binding to the TRPs with the exception of the TRPA1 C-terminus, where Sig-1R binding cooperates with calmodulin binding [55]. The authors of this study hypothesize that the binding of Sig-1R to TRPs may favor their open probability, while calmodulin would reduce TRP activity by competing and diminishing Sig-1R binding. Indeed, the calcium-activated calmodulin was previously shown to reduce calcium permeation through TRP channels [57,58,59,60]. Moreover, Sig-1R interaction with TRPs was regulated by steroids such as pregnenolone sulfate and progesterone, which by their action on the Sig-1R disrupted or promoted receptor-channel interactions [55]. Since Sig-1R is implicated in essential physiological processes, exploiting such interactions may represent a means to develop more selective and efficacious pharmacological interventions. In this context, it was recently shown that Sig-1R interaction with TRPV1 may reduce pain under physiological conditions such as pregnancy [56]. TRPV1 is considered as a key player in the transduction of noxious stimuli [61] and its inhibition has become an important pharmacological target for analgesic treatments [62]. Sig-1R directly interacts with TRPV1, thus regulating the number of TRPV1 channels localized at the plasma membrane. Progesterone is an endogenous ligand of this receptor, that displays the highest affinity for Sig-1R, as compared to the other steroid-type ligands [63]. Upon application of progesterone, the interaction of Sig-1R with the channel is disrupted, resulting in the downregulation of plasma membrane TRPV1 expression and, consequently, in a decrease in capsaicin-induced nociceptive responses of sensory neurons. These results were confirmed in vivo for both males treated with a synthetic antagonist of Sig-1R and pregnant females where progesterone levels were elevated [56]. In line with these results Sig-1R knockout mice exhibited endurance to pain and mechanical allodynia induced by formalin and capsaicin, respectively [64,65].

Another example of direct nongenomic steroid action on TRP regulation and their consequent physiological actions is the effect of testosterone on the TRPM8 channel. The TRPM8 channel is involved in prostate cancer progression, and has been proposed as a promising clinical target [66,67]. The channel expression increases during the initial stages of prostate cancer but is reduced after anti-androgen therapy [68]. Moreover, several studies have involved TRPM8 as a key player in cancer cell migration, and the transition to the androgen-independent aggressive stage of prostate cancer has been shown to positively correlate with loss of TRPM8 expression [66,69]. As the expression and/or activation of TRPM8 suppresses prostate cancer cell migration [69,70,71], TRPM8 was pinpointed as potential molecular target antagonizing metastatic transition of prostate cancer. Interestingly, we have recently shown that the androgen receptor interacts directly with TRPM8 channel with a subsequent inhibition of its channel activity [72]. More precisely, 10 nM testosterone application promotes accumulation of TRPM8 and androgen receptor proteins in cholesterol- and caveolin-rich fractions, while at higher (100 nM) testosterone concentration this preferential TRPM8/androgen receptor localization is lost and the androgen receptor is translocated to the nucleus. The decrease of TRPM8-mediated Ca^2+^ influx results in cell migration acceleration [72] and is in agreement with the reports of correlation between low serum testosterone level (<230 ng/dL or <8 nM) and tumor aggressiveness, poor prognosis, and PCa metastasis [73].

## 4. Capacitative Calcium Entry (CCE) and Associated Proteins

Store-operated calcium entry (SOCE) and the associated SOC/CRAC (Ca^2+^ release activated Ca^2+^) channels represent the main calcium entry pathway in nonexcitable cells [74]. While initially described in 1986 [75], the molecular nature of SOC/CRAC channels was only elucidated in 2005 with the discovery of its key components, namely Stim and Orai [76,77]. Stim1 represents the calcium-sensor detecting calcium level in the ER, and upon depletion of calcium stores, oligomerizes in regions of the ER near plasma membrane (i.e., ER-PM junctions) where it will directly interact with Orai1 channels, provoking their opening and the resulting capacitative calcium entry (CCE) allowing for refill of ER calcium stores [78]. In the last 15 years, many teams have investigated the nature and the stoichiometry of the interaction between Orai and Stim proteins. These studies have led to the current hypothesis that SOC/CRAC channels are comprised of hexamers of Orai1 channels, where Stim1 dimers interact with one or two Orai1 monomers, and to the identification of key regions in both proteins (for a review, see [74,78]). Adding a new level of complexity, it was also shown that several other proteins can interact with this complex, either directly or indirectly. Such an example are septins: while not directly interacting with the Orai-Stim complex, these proteins have been shown to play a key role in CCE control. Indeed, they will delineate lipidic microdomains through their interaction with membrane phospholipids (PIP2), and facilitate Stim1 translocation to ER-PM junctions, resulting in a general increase in Orai-Stim complex stability [79]. Another family of proteins widely studied are TRP channels. Indeed, in the 20 years following the initial description of SOCE, many TRP were presented as possible SOC channels (e.g., TRPC1, TRPC3, TRPV6). After the discovery of Orai-Stim, many studies aimed at identifying the exact nature of the relationship between TRP channels and this complex. It is currently proposed that TRP channels participate in the calcium influx elicited by depletion of ER calcium stores, but by forming distinct structures (e.g., Stim1-TRPC1 [80]). As these types of interaction have been already reviewed at length [78,81,82], in the following sections, we will briefly present several proteins directly interacting with the Orai1-Stim1 complex, and how they affect its function. For clarity, we will distinguish partners in two groups: those activating SOCE, and those exerting a negative effect on CCE.

**Figure 3:**
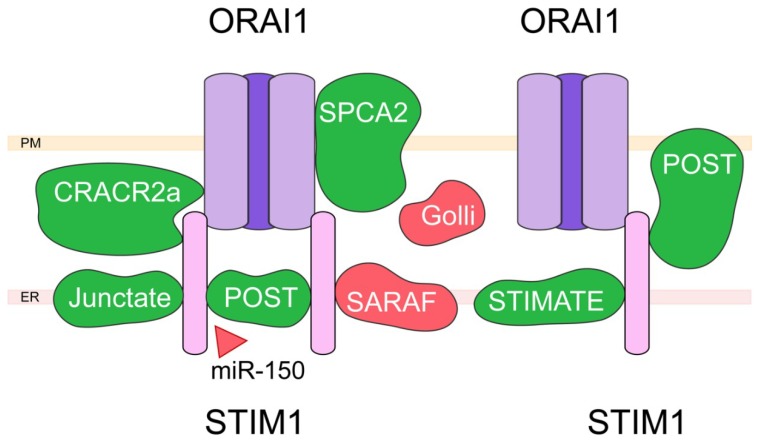
Targeting the store-operated calcium entry. Scheme depicting the Orai1 channel (in purple) and Stim1 (in pink) and their partner proteins. Activating partner proteins are shown in green, inhibiting partner proteins in red. Molecules targeting the partners are represented by triangles: red triangles indicate an inhibitory effect, green triangles an activating effect. PM, plasma membrane; ER, endoplasmic reticulum; CRACR2a, calcium release activated calcium channel regulator 2a; SPCA2, secretory pathway calcium ATPase 2; STIMATE, Stim-activating enhancer.

We will describe here five partner activating SOCE, namely the calcium release activated calcium channel regulator 2a (CRACR2a), Junctate, POST (TMEM20), secretory pathway calcium ATPase 2 (SPCA2) and Stim-activating enhancer (STIMATE). CRACR2a was initially described by Srikanth et al. in T cells as a cytoplasmic protein involved in the modulation of Stim1–Orai1 interaction [83] (Figure 3).

CRACR2a presents an EF-hand domain, and directly binds to the N-terminus domain of Orai1, most probably via the interaction with lysin 85 and lysin 87 on the channel. Indeed, mutation of these two residues into alanine leads to a dramatic decrease in Orai1-CRACR2a interaction. CRACR2a also directly binds Stim1 via its coiled-coil (CC) and proline/lysin rich sequence (PEST) domains. Moreover, while CRACR2a interacts with Stim1 and Orai1 at resting calcium concentration, the cytosolic calcium increase elicited by SOCE will lead to the dissociation of this complex, and the inhibition of SOCE. Junctate is a resident ER membrane protein identified by Srikanth et al. as a potential modulator of the Stim1–Orai1 interaction by promoting Stim1 recruitment at ER-PM junctions [84]. In this work, the authors identified the domains involved in this interaction, namely the N-terminus of Stim1 and the C-terminus of junctate (residues 71–236). They also showed that junctate presents a putative EF-hand domain (residues 77–88) important for the interaction with Stim1, and that this protein does not directly interact with Orai1. While the silencing of junctate led to a general decrease in SOCE, mutation in the EF-hand domain induced Stim1 punctae formation at the ER-PM junctions under resting condition, and to an increase in cytosolic calcium concentration. POST was identified during a screening searching for partner proteins of Orai1 in cells exposed to thapsigargin, an inhibitor of SERCA pumps inducing depletion of ER calcium stores [85]. It is suggested to belong to the drug/metabolite transporter superfamily, and presents 10 putative transmembrane domains. Despite its initial premise, this study has shown that POST interaction with Orai1 in the plasma membrane is not affected by store depletion. Indeed, POST was found to be mainly located in the ER under resting condition, store depletion leading to its translocation to ER-PM junctions where it colocalizes with Stim1. The same study found that POST promotes Stim1 interaction with several proteins following store depletion, namely SERCA2, PMCA, Na/K-ATPase, and exportin-1. It was also shown that silencing of POST leads to an increase in PMCA activity, leading the authors to postulate that ER calcium stores’ depletion could promote POST-Stim1 translocation to ER-PM junctions, resulting in PMCA inhibition, thus favoring cytosolic calcium increase. SPCA2 is closely related to SPCA1, whose role is to pump Ca^2+^ and Mn^2+^ inside Golgi lumen, thus enabling protein sorting, processing, and glycosylation. In their study, Feng et al. have shown that SPCA2 expression is increased in patients with breast cancer, and that its knock-down prevents cancer cell growth [86]. Moreover, they associated SPCA2 overexpression to a general increase in basal calcium level, this effect being independent from ER calcium release or its pump activity. Indeed, it results from the PM-localized SPCA2 capacity to directly bind Orai1 and activate it through a putative 2 steps mechanism: SPCA2 N-terminus (key amino acids: Valine 71, Threonine 75, Serine 78, and Valine 95) binds Orai1, promoting a conformational change in its structure leading to the exposure of its C-terminus that will also bind Orai1 and induce channel opening. This hypothesis has been since then confirmed by other groups, although the recent work of Smaardijk et al. has proposed that intracellular SPCA2 could be more relevant to Orai1 activation, while recognizing that SPCA2 localization in PM could be tissue dependent [87]. STIMATE (encoded by *TMEM110*) is an ER resident protein with multiple transmembrane domains. It was identified by Jing et al. as colocalizing with Stim1 under resting condition, and to follow Stim1 in punctae after store depletion [88]. STIMATE silencing was shown to reduce Stim1 punctae formation, as well as the resulting SOCE. The authors have proposed the following mechanism: STIMATE C-terminus directly binds Stim1 CC1 (coiled coil 1) domain, thus weakening the interaction between CC1 and SOAR (Stim1 Orai activating region) domains, and leading to Stim1 activation. Another study by Quintana et al. has shown that STIMATE could play a more general role by controlling the association between ER and plasma membrane, an effect involving its interaction with Stim proteins [89].

The partners inhibiting CCE are mainly Golli and SOCE-associated regulatory factor (SARAF). Golli MBP (myelin basic proteins) represent a family of proteins found in the brain, the thymus, and other immune organs. In T cells, the main Golli isoform is BG21. As shown by Feng et al., mice KO for BG21 exhibit a higher T cells activation rate [90]. While Golli is mostly found in the cytoplasm, it can be associated with the plasma membrane, a process involving a myristoylation site on the protein (Glycine 2). Moreover, mice KO for Golli exhibited increased CRAC activity, suggesting that Golli is an inhibitor of SOCE. While these results have been confirmed since then, and BG21 direct interaction with Stim1 C-terminus has been suggested [91], the underlying mechanism remains unknown. SARAF (TMEM66) is a 339 amino acids protein located in the ER membrane, with one putative transmembrane domain. As shown by Palty et al., overexpression of SARAF in HEK293 cells does not affect SOC current amplitude, but greatly increases its calcium-dependent inactivation [92]. This effect involves SARAF C-terminus domain that interacts with Stim1 in order to control SOCE, whereas its luminal N-terminus domain controls its inhibitory effect on the channel. According to this study, Stim1 and SARAF are associated under resting conditions, but store depletion increases their interaction in ER-PM junctions. Silencing of SARAF leads to more Stim1 being found at ER-PM junctions in the absence of any store depletion, an effect associated with elevated cytoplasmic calcium levels. While these authors propose that SARAF enhances Stim1 deoligomerization during store refilling, Jha et al. have shown that SARAF binds the Stim1 SOAR domain, and that when Stim1 is activated, the loss of interaction between the SOAR domain and SARAF leads to a fully activated Orai1 with no slow calcium dependent inactivation (SCDI) [93]. Since these initial works, SARAF has been proposed not only to interact directly with Orai1 and to promote its activation [94], but also to bind/modulate other calcium channels such as ARC, TRPC1, and TRPC6 (for a review, see [95]).

Since its initial discovery more than 30 years ago, SOCE has been at the center of many studies. However, with the identification of its molecular nature in 2005–2006, the complexity of SOCE regulatory mechanism has steadily increased, as shown above with a nonexhaustive list of SOCE main modulators. The next step will logically be to use this knowledge in order to target SOCE, and thus treat human patients afflicted with diseases linked to SOCE aberrant activity. In a recent study, miR-150 was shown as an endogenous inhibitor of POST, important for CD^8+^ T cells activation [96]. According to this work, targeting of SOCE partner protein POST with miR-150 could therefore represent a new treatment against auto-immune diseases. Since SOCE has been proposed to play a critical role in many human diseases, including cancer, its targeting is of the utmost importance [97,98]. However, due to the fact that SOCE is found in almost all cell types and organs, specific targeting of a given cell subtype presents a real challenge for any new potential treatment. In this context, the existence of partner proteins differentially expressed in different organs and tissues opens the door to new ways to specifically target SOCE in a given organ. More studies are however still required in order to identify specific drugs targeting these interactions.

## 5. Inositol 1,4,5-Trisphosphate Receptor (IP3R)

**Figure 4:**
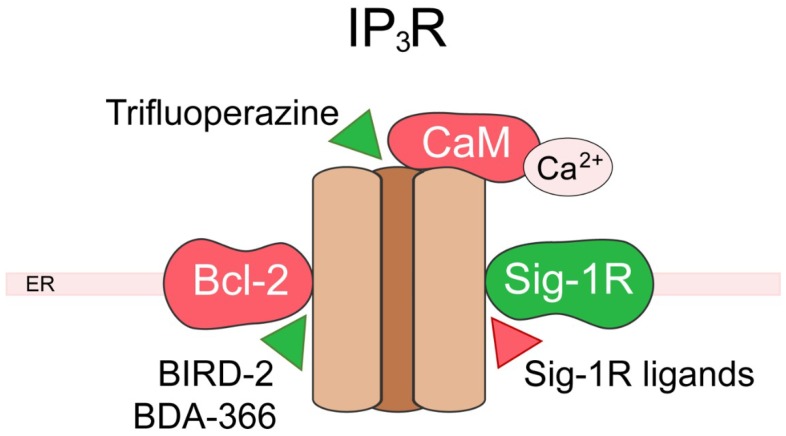
Targeting the inositol 1,4,5-trisphosphate receptor (IP3R). Scheme depicting IP3R and its partner proteins. Activating partner proteins are shown in green, inhibiting partner proteins in red. Molecules targeting the partners are represented by triangles: red triangles indicate an inhibitory effect, green triangles an activating effect. ER, endoplasmic reticulum; Bcl-2, B-cell lymphoma-2; Sig-1R, Sigma-1 receptor; BIRD-2, Bcl-2-IP3R disrupter-2.

The IP3Rs represent the main ER calcium-release channels in nonexcitable cells [99,100,101,102]. Mammalian cells express three distinct isoforms (IP3R1, IP3R2, and IP3R3) displaying different affinity to IP3 and are present in varying levels within different cell types. The calcium signaling resulting from IP3R plays a crucial role to regulate cell fate and its deregulation has been linked with several neurological disorders and cancers. As the major isoform in the human brain, IP3R1 has been extensively studied within the frame of brain pathology [103]. IP3R expression has also been shown to be altered in tumors, conferring increased survival to cancer cells. IP3R3 is overexpressed in glioma [104], gastric [105], and colorectal cancers [106], and mutated in head and neck squamous cancer [107]. IP3R2 has also been shown to be upregulated in diffuse large B-cell lymphoma [108]. In this section, we will develop IP3R regulation by two of its main partners, and their pathophysiological implications, namely B-cell lymphoma-2 (Bcl-2) in cancer and the Sig-1R in brain pathophysiology, as summarized in Figure 4.

The Bcl-2 protein family encompasses both pro- and anti-apoptotic proteins regulating the intrinsic mitochondrial pathway of apoptosis. Here, we will focus on the protein Bcl-2 who exerts its anti-apoptotic role in two ways [109]: (i) by binding and inhibiting pro-apoptotic proteins of the Bcl-2 family such as Bax and Bak at the mitochondria level; (ii) by binding and modulating IP3R at the ER level [110]. Bcl-2 has been shown to directly interact with IP3R, and multiple binding sites have been described [111,112,113,114]. Via these interactions, Bcl-2 directly modulates calcium release through the IP3R [115], thus promoting pro-survival calcium oscillations [116,117] and inhibiting pro-apoptotic calcium release [112]. Bcl-2 is therefore able to finely tune mitochondrial calcium uptake increasing ATP production and preventing mitochondrial calcium overload leading to cell death. Of note, Bcl-2 can also recruit proteins to act indirectly on IP3R [118]. Bcl-2 is upregulated in many cancers as a mean to escape cell death, its targeting, thus, represents an interesting anticancer strategy, and extensive efforts have been made to develop Bcl-2-ligands [119] (recently reviewed in [120]). Different classes of ligands have been developed, affecting Bcl-2′s effect on either IP3R, pro-apoptotic proteins, or both. Currently, the most promising peptide allowing for IP3R modulation through Bcl-2 is the Bcl-2-IP3R disrupter-2 (BIRD-2). As a BH4 mimetic, BIRD-2 targets the BH4 domain of Bcl-2, preventing its interaction with IP3R and inducing apoptosis through IP3R signaling [121,122]. Interestingly, a recent study showed that both ER and extracellular calcium content are crucial to determine the efficiency of BIRD-2, as it is able to switch IP3R signaling from pro-survival to pro-apoptotic [123]. BIRD-2 was able to trigger cell death in various cancer models such as diffuse large B-cell lymphoma [108,123,124], chronic lymphocytic leukemia [123,124,125], small cell lung cancer [126], and multiple myeloma [122,127]. In ovarian cancer, BIRD-2 was not able to kill cancer cells by itself but led to sensitization of tumor cells to chemotherapy through a Bcl-2-dependent pathway [128]. However, the efficiency of BIRD-2 seems to differ according to the expression levels of the different IP3R subtypes and could thus fluctuate between different cell types [108]. The BDA-366 small molecule was developed to mimic BIRD-2 function and overcome the limitations of the use of peptides as therapeutic drugs. This small molecule showed high affinity and selectivity for Bcl-2 [129] and induced cell death in lung cancer [129] and multiple myeloma [130]. However, BDA-366 was shown to act by disrupting the interaction of both Bcl-2-IP3R and Bcl-2-Bax. Moreover, this compound is able to induce cell death in cancer cells that do not express Bcl-2, showing the existence of a Bcl-2-independent mechanism. The other major class of peptides targeting Bcl-2 are BH3-mimetics that bind the hydrophobic cleft of Bcl-2, preventing its binding and inhibition of Bax and Bak pro-apoptotic proteins. These ligands have no effect on IP3R activity as shown in a recent study with Venetoclax (AB-199) [113]. Thus, through their double action on IP3R and pro-apoptotic proteins, Bcl-2 targeting shows interesting potential for anticancer therapeutics and one of the peptides has been approved to treatment [131]. To prevent excessive signaling, IP3R activity is negatively modulated by calcium. The calcium-binding calmodulin was shown to be able to bind and inactivate the receptor [132]. Trifluoperazine, an antipsychotic drug, has been shown to bind and inhibit calmodulin [133], leading to IP3R disinhibition and reducing glioma cell invasion [104].

Interestingly Sig-1R described above as a TRP channel partner protein, associates as well with IP3R. Upon ER stress, the ER resident Sig-1R can dissociate from binding immunoglobulin protein (BiP) to chaperone the IP3R in the mitochondria-associated membranes (MAM). There, the Sig-1R stabilizes IP3-activated IP3R preventing its degradation and allowing sustained calcium efflux towards the mitochondria to increase ATP production and maintain cell survival [49]. The chaperoning of IP3R by Sig-1R was shown to protect cells against stress in neurons [134,135,136,137,138] as well as cardiomyocytes [139,140]. Alteration of this mechanism has been linked with neurological disorders such as Huntington’s disease [141], amyotrophic lateral sclerosis (ALS) [142,143], and cancer [144,145]. In addition, sustained Sig-1R activation can lead to excessive calcium transfer to mitochondria leading to cell death as it was recently shown in brain endothelial cells [146]. Several splice variants and mutations of the S1R were identified leading to the loss of the ability to bind IP3R and sensitizing cell to apoptosis upon ER stress [147]. Among those Sig-1R variants, some have been linked with neurodegenerative disorders such as ALS [142,143]. The Sig-1R is targeted by many drugs that can be used to modulate IP3R calcium signaling. Indeed, the IP3R modulation by Sig-1R was shown to be reinforced by Sig-1R agonists and inhibited by antagonists in many different physiological and pathological cellular contexts [134,135,136,137,138,139,140,141,144,145]. Conversely, the Sig-1R can also modulate IP3R indirectly by stimulating IP3 production [148,149,150], increasing IP3R expression [151] and also by increasing Bcl-2 expression [152,153,154]. However, one needs to keep in mind that, as a chaperone protein, Sig-1R can bind and modulate other proteins upon ligand-stimulation and could thus alter calcium signaling in non-IP3R-related pathways (reviewed in [155]).

## 6. Conclusions

We have described here some of the recent advances in the characterization of partner proteins regulating calcium fluxes in the plasma membrane as well as in intracellular compartments, namely the endoplasmic reticulum and mitochondria. All the partners proteins of calcium channels, as well as the modulators of these interactions mentioned in the paragraphs here above are summarized in Figure 1, Figure 2, Figure 3 and Figure 4. While protein–protein interaction assays combined with mutagenesis techniques were crucial for the identification and characterization of ion channel-associated proteins, further insight is needed into 3D intermolecular dynamics of the interaction. As mentioned above, structural data were used as a basis in order to fully characterize some of the CaV2.2 [40] and ORAI [87] interactions. The structures of all calcium channels and receptors mentioned here were poorly exploited from the point of view of their partner proteins even though most of them have been resolved with different depth resolutions [156,157,158,159,160,161]. To date, in addition to the data from cryo-electron microscopy, that has emerged as one of the most effective techniques, advancements in structure-based computational tools also have to be used in order to fully understand the macromolecular membrane protein assemblies and their interactions. In this respect, tools such as homology modeling, docking and virtual screening, virtual amino acid scanning, and structure-based peptide design, can be coupled with the growing public database containing high resolution structures of ion channels and their partners for more efficient peptide development and drug discovery.

## Abbreviations

AAVis	Adeno-associated virus
ABP	AID-binding pocket
ALS	Amyotrophic lateral sclerosis
Bcl-2	B-cell lymphoma-2
BiP	Binding immunoglobulin protein
BIRD-2	Bcl-2-IP3R disrupter-2
CBD	Calcium channel binding domain
CC	Coiled-coil
CCE	Capacitative calcium entry
CRAC CRACR2a	Calcium release activated calcium channel Calcium release activated calcium channel regulator 2a
DHP	Dihydropyridines
ER	Endoplasmic reticulum
GK	Guanylate kinase
HVA	High-voltage activated channels
IP3R	Inositol 1,4,5-trisphosphate receptor
IP3R	Inositol 1,4,5-trisphosphate receptor
LVA	Low-voltage activated
MAM	Mitochondria-associated membranes
MBP	Myelin basic proteins
PEST	Proline/lysin rich sequence
PM	Plasma membrane
SARAF	SOCE-associated regulatory factor
SCDI	Slow calcium dependent inactivation
SCDI	Slow calcium dependent inactivation
Sig-1R	Sigma-1 receptor
SOC	Store-operated calcium
SOCE	Store-operated calcium entry
SPCA2	Secretory pathway calcium ATPase 2
Stim STIMATE	Stromal interaction molecule Stim-activating enhancer
TRP	Transient receptor potential
VDCC	Voltage-dependent calcium channels
